# Gliomatosis peritonei: a series of eight cases and review of the literature

**DOI:** 10.1186/s13048-016-0256-5

**Published:** 2016-07-29

**Authors:** Dan Wang, Cong-wei Jia, Rui-e Feng, Hong-hui Shi, Juan Sun

**Affiliations:** 1 Department of Obstetrics and Gynecology, Peking Union Medical College Hospital, Chinese Academy of Medical Science and Peking Union Medical College, Shuaifuyuan NO.1, Dongchen District, Beijing, 100730 People’s Republic of China; 2 Department of Pathology, Peking Union Medical College Hospital, Chinese Academy of Medical Science and Peking Union Medical College, Shuaifuyuan NO.1, Dongchen District, Beijing, 100730 People’s Republic of China; 3 Department of Obstetrics and Gynecology, Maternal and Child Health Care Hospital of Zaozhuang, Wenhua Road, Shizhong District, Zaozhuang, 277100 People’s Republic of China

**Keywords:** Ovarian teratoma, Gliomatosis peritonei, Prognosis

## Abstract

**Background:**

Gliomatosis peritonei (GP) is a rare condition characterized by mature glial tissue implants widespread in the peritoneum. The GP is often associated with ovarian teratoma. However, little is known about the characteristics and prognosis of GP. The purpose of this study was to describe the features, treatment, and prognosis of GP. Additionally, we review previously reported cases of GP, summarizing the presently known data.

**Methods:**

From January 2000 to January 2016, cases of ovarian teratoma and GP treated at Peking Union Medical College Hospital were reviewed. We assessed the pathology, treatments, and outcomes along with prognostic information. Additionally, the literature regarding this clinical condition was also reviewed.

**Results:**

Eight patients met the inclusion criteria. Patients had a median age of 20 (range, 15–25) years. GP was diagnosed as the primary tumor in 6 patients and at a secondary surgery in two patients. The primary ovarian tumor consisted of immature teratoma (*n* = 7) and mature teratoma (*n* = 1). Grades of immature ovarian teratoma were 2, grade 1; 3, grade 2; and 2, grade 3. Tumors mean had a size of 20.4 (range, 11–30) cm. The median follow-up time was 60.5 (range, 3–144) months. All cases had conservative surgery and seven of them had macroscopic residual disease postoperatively. During the study period, the eight patients remained alive and asymptomatic. Three patients in the study experienced spontaneous pregnancy. After reviewing the existing literature, a total of 14 patients with nodal gliomatosis were present and 10 of them were alive. According to the literature review, five articles reported more than five cases. Of a total of 67 patients, 60 of them remained alive.

**Conclusion:**

The prognosis of immature ovarian teratoma with GP is favorable. Complete resection of GP is often difficult. Residual peritoneal disease in GP can be asymptomatic and quiescent over a long period. A more conservative surgical approach may be carried out in patients with massive peritoneal spread after the presence of metastatic immature elements is excluded. Owing to the risk of recurrence and malignant transformation of GP, a long-term follow-up is necessary for patients with residual peritoneal disease.

## Background

Gliomatosis peritonei (GP) is a rare disease characterized by many peritoneal and omental implants composed of mature glial tissue. It is mainly associated with ovarian teratomas, especially in immature ovarian teratoma. Thus far, only about 100 cases have been reported [1]. According to the WHO grading system for immature ovarian teratoma, GP is considered as grade 0. In fact, GP has long been associated with good prognosis. Because of its rarity, the prognostic effect of GP and its clinical characteristics remain to be clarified. In the present study, we report eight cases of GP diagnosed and treated at our hospital. We also reviewed the relevant literature to increase the understanding of this disease.

## Methods

From January 2000 to January 2016, cases of ovarian teratoma with GP diagnosed at Peking Union Medical College Hospital were reviewed. Medical and pathological records were retrospectively reviewed to evaluate the clinical features of all patients. Patients were included in the study if they met the following criteria: histological review of the first tumor by an expert pathologist; diagnosis of ovarian teratoma (mature or immature), excluding other malignant germ cell tumors; For patients who received adjuvant chemotherapy after surgery, immature teratoma (IMT) associated with GP at the secondary surgery were excluded; patients whose follow-up information was intact. The relevant literature was reviewed simultaneously.

Ovarian teratomas were graded according to the published guidelines. Peritoneal nodal specimens were carefully examined to exclude the co-existence of metastatic immature elements. The precise surgical details were recorded. Oncologic and pregnancy outcome were analyzed.

All patients had undergone conservative surgery (conservation of uterus and at least a portion of an ovary). Additional surgical procedures, such as peritonectomies, appendectomy, omentectomy, and lymphadenectomy were performed depending on the stage and macroscopic situation during the operation. Some patients underwent a second surgery.

Adjuvant chemotherapy was given because of tumor rupture, stage IC, or advanced stage or high grade of teratoma (grade 2 or grade 3) according to the NCCN guideline. Follow-up was based on clinical examination and radiologic imaging. Patient information was updated until April 2016 with a median follow-up of 60.5 (range: 3–144) months.

## Results

**Fig. 1:**
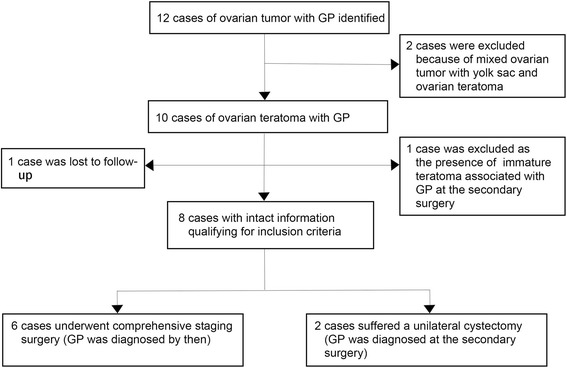
Summarized figure of ovarian teratoma with GP

**Fig. 2:**
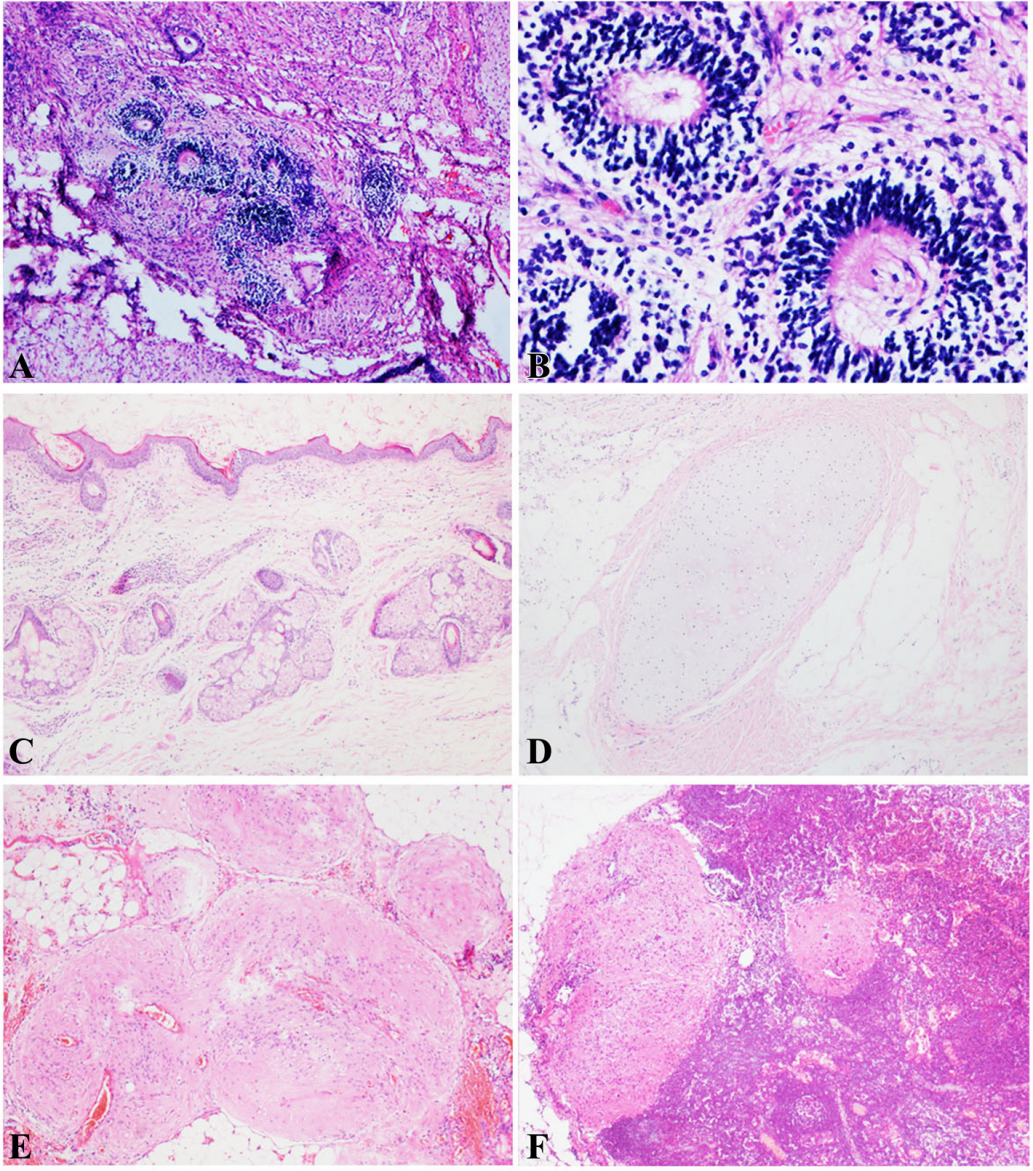
Immature neuroepithelial tissue in the form of neuroectodermal rosettes are admixed with mature tissues **a** HE stain × 40. Immature neuroectodermal tissue **b** HE stain × 200. Ovarian mature teratoma is composed of various tissue components like skin and skin appendages **c**, cartilage **d** HE stain × 40. Peritoneal gliomatosis showing a discrete nodule of mature glial tissue, which surrounded by fibroadipose tissue of peritoneum **e** HE stain × 40. Mature glial tissue is seen in right iliac lymph node **f** HE stain × 40

During the study period, eight patients that presented at our hospital met the inclusion criteria (shown in Fig. 1). The patients’ clinical and pathological features are presented in Table 1. Patients had a median age of 20 (range: 15–25) years. The mean size of tumors was 20.4 (range: 11–30) cm. In the present study, seven primary ovarian tumors were immature teratomas (*n* = 7; grade 1, 2; grade 2, 3; and grade 3, 2) (Fig. 2 a, b), and one was a mature teratoma (*n* = 1) (Fig. 2c, d). The level of blood CA125 before therapy was available in seven patients, with a median CA125 level of 340.3 U/ml (range: 98.9–673.5 U/ml).

**Table 1:** Clinical and pathological features of 8 cases with GP

case	Age, y	CA125 U/ml	Tumor Size (cm)	Surgical Procedure (1st surgery)	Primary tumor	Metastatic Tissue of the 1st surgery	Adjuvant therapy	GFAP	Residual GP	Outcome
1	23	420.1	18	USO, Peritonectomies, Appendectomy, omenctomy	IMT, G1	GP	PEB*3	NA	YES	Alive 16 months
2	25	98.9	22	USO, Peritonectomies, Omenctomy, lymphadenectomy	MT	GP, Nodal, gliomatosis	NO	+	YES	Alive 3 months
3	16	340.4	20	USO, Peritonectomies, Omenctomy, lymphadenectomy	IMT, G2	GP, Nodal, gliomatosis	PEB*6	+	YES	Alive 68 months
4	18	171.7	14	cystectomy	IMT, G2	NA	PEB*3	NA	YES	Alive 38 months
5	23	NA	11	cystectomy	IMT, G2	NA	NO	NA	YES	Alive 61 months
6	22	381.1	25	USO, Peritonectomies, Omenctomy, lymphadenectomy	IMT, G1	GP, Nodal, gliomatosis	NO	+	YES	Alive 60 months
7	15	673.5	23	USO, Peritonectomies, Omenctomy, lymphadenectomy	LOV: IMT, G3, ROV: G1	GP	PEB*2 PVB*2 PV*2	NA	NO	Alive 97 months
8	17	238.7	30	USO, Peritonectomies, Omenctomy, Lymphadenectomy, Appendectomy	IMT, G3	GP, Nodal, gliomatosis	PEB*4	NA	YES	Alive 144 months

*GFAP* glial fibrillary acidic protein, *GP* gliomatosis peritonei, *IMT* immature teratoma, *MT* mature teratoma, *NA* not available, *USO* unilateral salpingo-oophorectomy, *PEB* bleomycin, etoposide, cisplatin, *PVB* bleomycin, vincristine,cisplatin, *PV* vincristine,cisplatin

Six patients had undergone comprehensive staging surgery, which led to the diagnosis of GP (Fig. 2e). The other two patients underwent unilateral cystectomy as the first surgery and GP was detected in the secondary surgery (cases 4 and 5). Seven patients had residual disease postoperatively.

In case 4, the patient presented with abdominal distension, abdominal pain, nausea, and vomiting. The B ultrasound showed a 14-cm cystic-solid pelvic mass. She underwent a unilateral cystectomy and was diagnosed with immature ovarian teratoma (grade 2). Subsequently, she received three cycles of bleomycin, etoposide, and cisplatin (PEB). Four months after the completion of chemotherapy, the computed tomography (CT) showed masses in Douglas’ pouch and on the liver surface with enlarged pelvic lymph nodes. All tumor markers were negative before the second surgery. Second surgery showed numerous miliary nodules on the surface of the intestine and masses of about 3 to 5 cm in Douglas’ pouch. The implants were so dispersed that complete resection of the tumors was impossible. Frozen section of the mass in Douglas pouch showed mature glial elements without immature tissues. Thus, growing teratoma syndrome (GTS) was diagnosed. After the diagnosis of GP, she did not receive further therapy. She was remained alive with persisting but asymptomatic disease 32 months after the diagnosis of GP (36 months after the diagnosis of immature ovarian teratoma).

In case 5, the patient underwent laparoscopic cystectomy because of ovarian cyst at another hospital. The pathologic evaluation indicated the presence of immature teratoma (grade 2). However, she was not given chemotherapy at that time. Six months after the first surgery, she suffered from recurrence of the ovarian cyst. A second laparoscopic surgery was performed, revealing both ovarian cysts, as well as peritoneal and pelvic wall implants. Laparoscopic cystectomy was performed. Surgical pathology showed mature teratoma, associated with GP. Ten months later, a CT showed multiple masses in the abdominopelvic cavity, near the liver and spleen. The surgical procedure consisted of a suboptimal cytoreduction. After the second surgery, the pathologic evaluation of resected tissue showed mature teratoma and mature peritoneal gliomatosis. She remained alive and asymptomatic 53 months after the diagnosis of GP (61 months after the diagnosis of mature ovarian teratoma).

Four cases (case 2, 3, 6, and 8) of nodal gliomatosis were detected in form of glial tissue in the pelvic lymph nodes (Fig. 2f). A summary of the cases of nodal gliomatosis is presented in Table 2. A total of 14 patients with nodal gliomatosis were described and 10 remained alive at the time of the last follow-up. Most of the published papers on GP were case reports. Only 5 articles in the literature reported more than five cases (Table 3). For a total of 67 patients, 60 of them were still alive at the time of the last follow-up.

**Table 2:** Summary of nodal gliomatosis cases reported in the literatures

Authors	Age	LN sites	Primary tumor	Treatment	Outcome
Benirschke [16], 1960	18 ys	Retroperitoneal,ilac,cervical axillary	Mature teratoma	chemoradiotherapy	Dead 8 months
Nagashima [13], 1974	22ys	Inguinal, mesenteric, mediastinal, cervical	IMT	S+ Ch	Dead 8 months
Shafie [14] 1984	12 ys	Omental	MT	S+ Ch	NR 5 ys
Perrone [15] 1986	10 mo	Para-arotic	IMT G1	Surgery	NR 9 months
Khan [17] 2005	23 ys	Lymph node	IMT G1	S+ Ch	NA
Fang [18] 2015	20 ys	Para-arotic	IMT G3	S+ Ch	Alive 36 months
Kim [6] 2013	34 ys	Hypogastric	IMT G1	S	NR 9 months
Li Liang [8] 2015	18 ys	Lymph node	IMT G1	NA	ANED 19 months
	42 ys	Lymph node	MGCT	NA	AWD 23 months
	10 ys	Lymph node	MGCT	NA	ANED 11 months
Present study	25 ys	iliac	MT	Surgery	Alive 3 months
	16 ys	Iliac	IMT G2	S+ Ch	Alive 68 months
	22 ys	iliac	IMT G1	S+ Ch	Alive 60 months
	17 ys	iliac	IMT G3	S+ Ch	Alive 144 months

*ANED* alive with no evidence of disease, *AWD* alive with disease, *Ch* chemotherapy, *IMT* immature teratoma, *LN* lymph node, *MT* mature teratoma, *MGCT* mixed germ cell tumor, *NA* not available, *NR* not recurrence, *S* surgery, *ys* years

**Table 3:** Cases of ovarian teratoma associated with GP in studies that reported more than five cases

Authors	Cases	Median Age ys	Ovarian neoplasm	Diagnosis	Recurrence	Treatment	Follow up
Norris [10], (1976)	7	17	IMT: G1: 5, G2-G3: 2	1st surgery: 7	NA	S: 4, S + Ch: 1, S + Rx:2	5 alive, 1 dead, 1 NA
Harms [11], (1989)	13	11.5	IMT: G1:8, G2-G3: 5	1st surgery: 11, 2nd surgery: 2	NO	S: 6, S + Ch: 7	13 alive
Yoon [2], (2012)	16	13	IMT: G1: 4, G2-G3: 11, MT:1	1st surgery: 15, 2nd surgery: 1	37.5 %, (6/16)	S: 3, S + Ch: 13	15 alive, 1 dead
Bentivegna, [12], (2015)^a^	9	36	IMT: G1: 5, G2-G3: 4	1st surgery: 1, 2nd surgery: 8	22.2 %, (2/9)	S: 5, S + Ch: 4	9 alive
Liang [8], (2015)	14	NA	IMT: G1: 5, G2-G3: 9	1st surgery: 10, 2nd surgery: 4	NA	NA	10 alive, NA: 4
Present	8	20	IMT: G1: 2, G2-G3: 5, MT: 1	1st surgery: 6, 2nd surgery: 2	NO	S: 3, S + Ch: 5	8 alive
Total	67	NA	IMT: G1: 29, G2-G3: 35, MT: 2	1st surgery: 50, 2nd surgery: 17	17.4 %, (8/46)^b^	S: 21, S + Ch: 30, S + Rx:2, NA:14	60 alive, 2 dead, NA: 5

*Ch* chemotherapy, *IMT* immature teratoma, *MT* mature teratoma, *NA* not available, *S* surgery, *Rx* radiotherapy

^a^One case in the article (case 8) was consisted of mixed ovarian germ tumor (yolk sac and dysgerminoma and mature teratoma). Thus, the table shows 9 cases

^b^As there was no data available in the reference 8 and 10, we just add up the data from the remaining articles

**Fig. 3:**
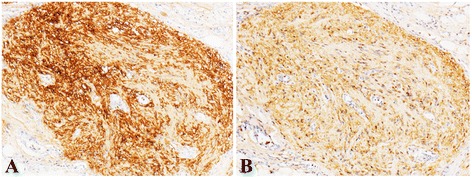
Positive immunohistochemical staining of the mature glial tissue with glial glial fibrillary acidic protein immunostain (**a**) and S 100 (**b**) × 100

Five patients were given chemotherapy (PEB or bleomycin, vincristine, and cisplatin [PVB]). If the dose of bleomycin reached the lifetime dose, the chemotherapy regimen was changed to PE or PV by omitting bleomycin. Immunohistochemical staining for glial fibrillary acidic protein (GFAP) was available in three cases. All three cases were positive for GFAP (Fig. 3a). Two of them were positive for S-100 protein (Fig. 3b). Three patients (cases 5, 6, and 8) in our group experienced a spontaneous pregnancy.

## Discussion

The metastatic implantation of mature glial tissue on the surface of the peritoneum, omentum, and abdominal lymph nodes is defined as GP. In a clinical setting, these widespread peritoneal, grayish tan-colored, tiny nodules encountered intraoperatively may be misdiagnosed as ovarian carcinomas or peritoneal tuberculosis. Ideal optimal resection may not be achieved in such cases with widespread implantation. However, GP has a favorable prognosis. In the report by Yoon et al., the overall survival did not differ between immature ovarian teratomas with GP and immature ovarian teratomas without GP, though patients with GP presented more frequent recurrence and shorter recurrence-free survival [2]. All patients (15 cases with GP) except one are currently alive when the articles were published.

GP is often diagnosed on HE-stained tissue sections, and its differentiation with low-grade epithelial ovarian tumors was difficult. A positive staining reaction of glial tissue for the neural marker, GFAP, is helpful. GFAP immunostain confirmed the glial nature of the tissue. A strong expression often suggests tumor cells are mature and well differentiated [3].

The etiology of GP is largely unknown. According to previous reports, there are two theories about the development of GP. One relates to capsular defects of the primary teratoma or dissemination via angiolymphatic channels. In some cases, GP within the omentum immediately adjacent to a capsular defect supports the mechanism of spread in its inception [4]. In 11 of the 12 cases in Robboy’s report, the capsule either had a tear or was adherent to the omentum or adnexal structure [5]. In support of lymphatic dissemination, mature glial tissue has been presented in para-aortic and pelvic lymph nodes with or without the presence of GP (Table 2). In some reports, GP was only found in the second surgery (as in case 4 in the present study). Kim et al. reviewed about 100 cases in the literature and found 9 cases of nodal gliomatosis in the pelvic or para-aortic lymph nodes with or without the presence of GP [6]. However, in their literature review, there were three cases of nodal gliomatosis presented as grades 1 to 3. As the nodal implantation contained immature elements, these should not be considered as nodal gliomatosis but as metastasis of the immature teratoma.

The other theory suggests that glial foci are genetically unassociated with teratoma. In Ferguson’s study [7], they use polymorphic microsatellite (MS) loci in two cases to address the origin of GP. They found that glial implants and normal tissue showed heterozygosity, while the teratoma showed homozygosity at the same MS loci. Their findings indicated that glial implants in GP were unrelated to the ovarian teratoma and arise from normal cells such as pluripotent Müllerian stem cells. It is possible that peritoneal stem cells can differentiate into glial cells under the stimulation of some factors secreted by teratomas [8]. The occurrence of GP after ventriculoperitoneal shunt operations when glial tissue is transported from cerebrospinal fluid into peritoneal cavity via shunt further support this theory [9]. However, the detailed mechanisms by which subperitoneal cells develop into glial follicles remain to be determined.

The stage and grade of the primary teratoma and the grade of its metastatic tumor are related to the prognosis of teratoma. Robboy and Scully reviewed the literature and presented a large series of cases [5]. They found that the prognoses in metastasized ovarian teratomas, or in ovarian teratomas leading to peritoneal implants, are favorable when the implants are composed of fully mature glial tissue. Müller reviewed all cases of GP published between 1906 and 2002. Eleven cases showed adverse outcomes [1]. They found that the recurrence of disease was associated with lack of extensive histological sampling at the first surgery. Thus, all specimens need to be adequately sampled and multiple biopsies should be taken to exclude immature glial tissue or teratoma elements. Once the presence of immature teratoma is confirmed in the metastatic tissue, the treatment scheme and prognosis may change.

Most of the papers on GP are cases. At present there are only 5 articles which report more than five cases (Table 3) [2, 8, 10–12]. Most cases of GP are associated with ovarian teratoma, especially with immature ovarian teratoma. In Yoon’s report, there were a total of 16 ovarian teratomas associated with GP [2]. Among them, 15 cases were of immature ovarian teratomas of various grades (4 cases were grade 1; 11 cases were grade 2 or 3). Liang reported the largest series of GP cases so far [8]. Sixteen of 23 cases were associated with immature ovarian teratomas. However, immature ovarian teratoma with GP showed better prognosis than would be expected based on the grading of the primary immature teratoma. In Norris’s report, the survival of patients with grades 1, 2, and 3 were 82 %, 63 %, and 30 %, respectively [10]. In Yoon’s report, all but one case of immature teratoma with GP remained alive when their report was published, although GP showed more frequent recurrences [2]. In Robboy’s report, 12 patients were alive and well [5]. More over, the survival of 8 patients in our series may also indicate that the presence of mature glial implants does not affect adversely the prognosis of ovarian teratoma.

Presence of glial tissue in lymph nodes is rare. Thus far, 14 cases have been reported in nine articles [6, 8, 13–18] (Table 2). Based on previous reports, adjuvant chemotherapy is unnecessary for patients with retroperitoneal lymph node metastasis of mature glial tissue. The prognosis of patients with nodal gliomatosis is favorable. In our series, patients with nodal gliomatosis remained alive and well during the follow-up period.

Sometimes immature ovarian teratoma can be associated with miliary spread of immature implants. After surgery and chemotherapy, the immature tissue may transfer into mature tissue. Chemotherapeutic retroconversion has also been called growing teratoma syndrome (GTS) [19]. However, there are some differences between GP and GTS. First, GP is composed of pure mature implants in the peritoneum without other mature nonglial tissues which can be seen in GTS. Second, GP could be encountered in the first surgery without prior chemotherapy. At last, the management of these two terms is quite different. An optimal cytoreduction is recommended for GTS to avoid complications such as bowel obstruction and perforation [20]. Because the lesions in GP are extensive, complete resection of GP is usually difficult. Luckily residual peritoneal disease in GP can be asymptomatic and quiescent over a long period [12]. Thus, residual implants can be ignored and the therapy is mainly depended on the stage and grade of the primary ovarian teratoma. Seven cases in our series presented macroscopic residual disease at postoperatively, and all of these patients remained alive and asymptomatic during the median follow-up of 60.5 months.

Bentivegna reported 10 cases of GP, while six of them were managed by conservative surgery [12]. All of their patients were asymptomatic at the time of the last consultation, and half had incomplete resected macroscopic GP. These authors found residual peritoneal disease could be totally quiescent over a long period without any impact on patient outcomes. As ovarian teratoma mostly occurs in young women who wish to preserve fertility, it is important to reduce the surgical scope and reduce surgical trauma without compromising cure rate. As residual implants of GP can be ignored, a more conservative surgical approach may be carried out in patients with massive peritoneal spread after exclusion of the presence of metastatic immature elements.

In the review of literature undertaken by Müller (86 cases plus their own two cases) [1], three progressions were observed: GP was asymptomatic and detected by a secondary surgery; GP may undergo “fibroblastic transformation” and gradually disappear [21]; GP may transform into malignant glial neoplasms such as glioblastoma which can result in patient death [22]. In most of the cases, patients with massive peritoneal spread cannot be completely resected, but these lesions can be totally quiescent over a long period. GP is associated with frequent recurrence in patients with immature ovarian teratoma [2]. In rare cases, GP showed potential for malignant transformation after several years. Thus, a long period of careful monitoring for patients with GP may be needed.

## Conclusion

Gliomatosis peritonei is metastatic implantation of mature glial tissue on surfaces of peritoneum. It is often associated with ovarian teratoma of any grade. The prognosis of GP is favorable. Because GP is always present with massive peritoneal implantation, optimal resection is difficult. Although residual peritoneal disease can be totally quiescent over a long period, long-term follow-up is needed for patients with residual disease. A more conservative surgical approach may be carried out in patients with massive peritoneal spread.

## Abbreviations

GFAP: glial fibrillary acidic protein; GP: Gliomatosis peritonei; GTS: growing teratoma syndrome; IMT: immature ovarian teratoma; MGCT: mixed germ cell tumor; PEB: bleomycin, etoposide, and cisplatin; PVB: bleomycin, vincristine, cisplatin; USO: unilateral salpingo-oophorectomy.
